# No Sex‐Differences in Learning Trap‐Gap Problems in Zebra Finches

**DOI:** 10.1002/ece3.72440

**Published:** 2025-11-10

**Authors:** Connor T. Lambert, Benjamin A. Whittaker, Brandon Neil, Cailyn Poole, Andrés Camacho‐Alpízar, Julia L. Self, Sara C. Blunk, Lauren M. Guillette

**Affiliations:** ^1^ Department of Psychology University of Alberta Edmonton Alberta Canada

**Keywords:** cognitive ecology, learning, nest building, physical cognition, sex‐differences, zebra finch

## Abstract

Sex differences in cognition are often predicted based on ecological roles, particularly when one sex engages more extensively in specific behaviors that might be subject to selective pressure. In zebra finches ( *Taeniopygia guttata* ), males choose and deposit the majority of the material into the nest and might therefore exhibit enhanced physical cognition. We tested this hypothesis using the trap‐gap task, a modified shape–frame matching paradigm designed to evaluate how animals assess object–hole relationships. In this task birds pulled food‐containing trays attached to strings through gaps in barriers. Birds were trained on either a barrier task (choosing the correct gap size to fit a tray between barriers with different gaps) or a tray task (choosing the correct tray size between barriers with the same gap), then transferred to the alternate task (called the transfer test). Contrary to predictions, males and females showed no differences in the number of trials to reach learning criteria or in the number of errors in the transfer test. Birds required more trials, on average, to learn the barrier task compared to the tray task, and the transfer test was at chance, suggesting birds relied on absolute cue‐based strategies rather than learning the object–gap relationship (relative cue‐based strategies). These findings align with previous research showing no sex differences in learning about material properties in zebra finches, despite males' dominant role in nest building. The lack of sex differences in performance may stem from a mismatch in spatial frames of reference: while nest building relies on an egocentric (body‐centered) frame, the trap‐gap tasks uses an allocentric (object‐centered) frame. Our findings highlight the complexity of linking behavioral sex roles to cognitive specialization and underscore the importance of task design and ecological relevance in comparative cognition research.

## Graphical abstract

This study examined whether male zebra finches, which play a larger role in nest building, show superior physical cognition compared to females using trap‐gap tasks. Contrary to expectations, no sex differences were found in learning or task transfer performance, and most birds relied on simple cue‐based strategies rather than object–gap relationships. The results suggest that cognitive specialization may not align directly with sex‐based behavioral roles and highlight the importance of ecological relevance in cognitive testing.

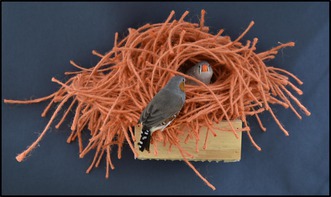

## 1 Introduction

One approach to understanding how evolution shapes cognitive abilities is to investigate among‐individual variation in behavior related to ecologically relevant tasks—especially differences between sexes where natural selection likely exerts different pressures on males and females of the same species. One of the most well‐supported hypotheses in this area concerns sex differences in home range size and spatial cognition (Jones et al. 2003). This hypothesis predicts that in species where males occupy larger home ranges than females, males will exhibit superior spatial cognitive abilities due to the increased demands of navigating larger territories. Empirical support for this prediction comes from various spatial cognition tasks, such as the Tolman sunburst maze (Gaulin and FitzGerald 1986), symmetrical maze (Gaulin and FitzGerald 1989), and Morris water maze (Kavaliers et al. 1998). Male meadow voles ( *Microtus pennsylvanicus* ), a polygynous species, have a larger home range than females and also outperform females on these tasks (Kavaliers et al. 1998). In contrast, this pattern is not observed in monogamous species like the prairie vole ( *Microtus ochrogaster* ) or the pine vole ( *Microtus pinetorum* ) where home range sizes do not vary between the sexes (Kavaliers et al. 1998).

Similarly, in brood‐parasitic birds, selection pressures differ markedly between the sexes. Females first locate and then remember the locations of host nests and monitor the developmental stage of each nest (e.g., egg‐laying, incubation) throughout the breeding season (Guigueno and Sherry 2017). These behaviors require both spatial memory and numerical ability. Reflecting these demands, female brown‐headed cowbirds ( *Molothrus ater* ) and shiny cowbirds ( *Molothrus bonariensis* ) outperform males on tests of large‐scale spatial ability (Guigueno et al. 2014; Lois‐Milevicich et al. 2021) and numerical discriminations (Guigueno et al. 2025).

Nest building is another reproductive behavior where natural selection may exert different pressures on males and females of the same species. Specifically, the sex responsible for building may exhibit enhanced physical cognitive abilities compared to the non‐building sex: that is, the ability to acquire, use, and understand information about the physical properties of the environment (Auersperg et al. 2017). Nest‐building behavior is a useful model examining the evolution of cognition (Guillette and Healy 2015; Healy et al. 2023), as it enables cross‐species comparisons in which nest construction is performed solely by the male, solely by the female, varies with nesting stage, or is shared by groups (Sheard et al. 2023). The nest‐building process generally involves a series of ecologically important decisions, including where to build, when to begin building, and with what materials. The cognitive demands of nest building likely change as construction progresses: the builder must select suitable materials, transport them to the nest site, and manipulate them to construct and shape the growing nest—a dynamic physical cognition problem to solve.

Attempts to investigate sex differences in the cognitive processes underlying nest‐building decisions in zebra finches (*Taenopygia guttata*), where males are primarily responsible for selecting and depositing nesting material (Goodwin 1982; Zann 1996), have yielded results that contradict our predictions. It was hypothesized that sex‐specific selection pressures on physical cognitive abilities, due to the males' dominant role in nest building, would lead to males learning to discriminate between materials with different physical properties (e.g., short vs. long, stiff vs. flexible) and remember these discriminations more readily than females. However, this prediction was not supported. A series of three experiments employed a foraging board task in which birds had to pull string with specific properties in order to remove chips covering wells and access food rewards (Lambert et al. 2021). These three studies found similarities in learning between female and male zebra finches, suggesting no sex differences in physical cognition with respect to discriminating material properties. To gain broader insight into whether sex differences exist in physical cognition between the building and non‐building sex, here we test a different paradigm that is also ecologically relevant for nest construction: the trap‐gap problem, which is a version of the shape–frame matching task.

**FIGURE 1:**
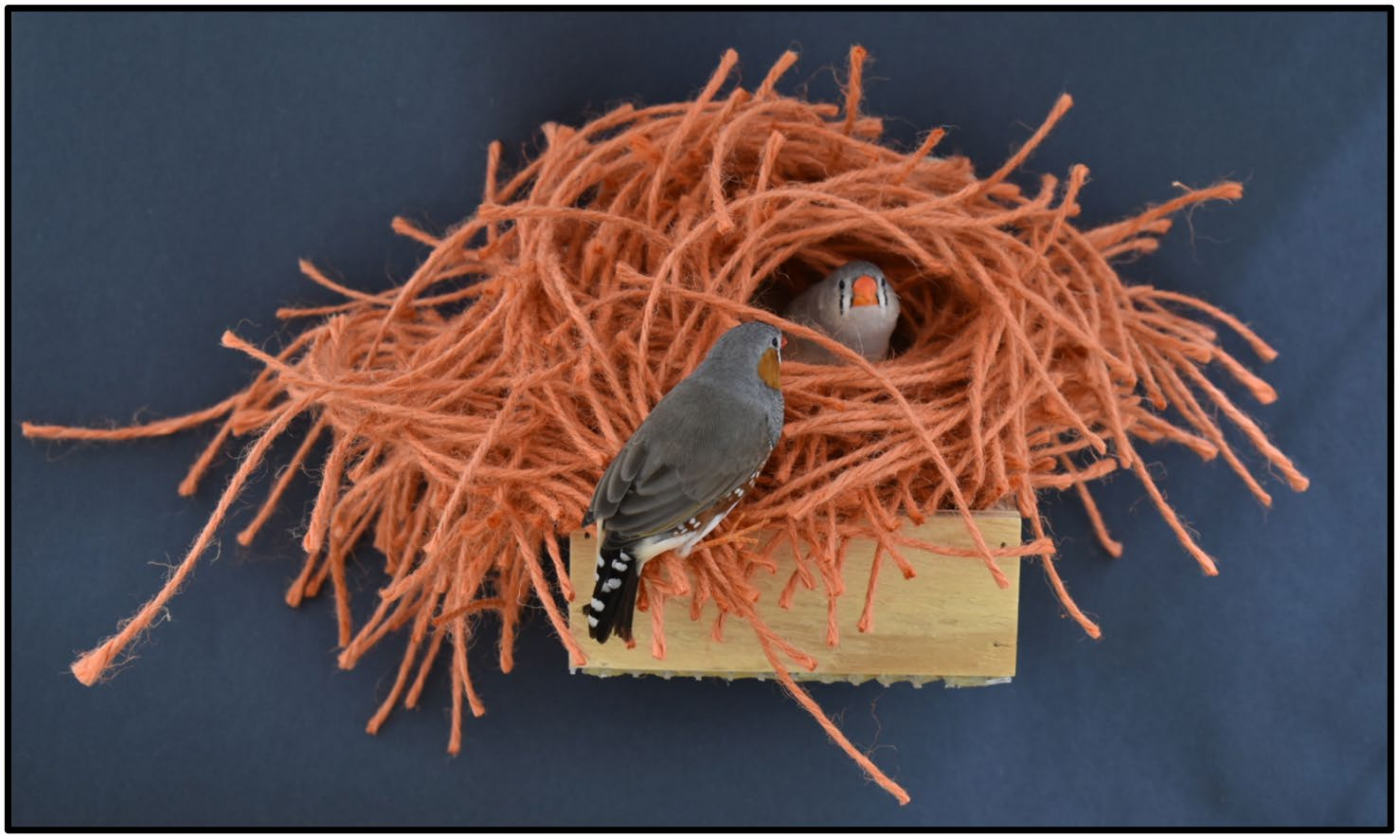
Example of a zebra finch nest built in our laboratory using 15 cm lengths of orange jute twine, with the male on the left and the female on the right in the nest entrance. This domed structure closely resembles the nests that zebra finches build in the wild using twigs and grasses.

One way to explore how animals understand physical relationships between objects is through the shape‐frame matching task, which requires fitting an object through a correspondingly shaped hole. This task is particularly relevant to the nest‐building behavior of male zebra finches, who construct their dome‐shaped nests from the inside out, both in the wild and in the laboratory. This method of construction showcases zebra finch males' ability to perform shape‐frame matching, as it involves learning how to fit building materials into the structure of a partially completed nest (Figure 1, Supporting Information: video). While shape‐frame matching tests are commonly used with mammals that have opposable thumbs, allowing them to manipulate shaped blocks into matching holes (e.g., Fragaszy et al. 2011; Örnkloo and Von Hofsten 2007), the task has also been adapted for other species. For example, parrots have been tested using a modified version, called the trap‐gap problem, where they pull strings attached to trays of varying sizes to determine whether they fit through specific gaps (van Horik and Emery 2016).

In this experiment, we investigated potential sex differences in physical cognition in zebra finches using the trap‐gap test (van Horik and Emery 2016), a modified ecologically relevant version of the shape‐frame matching task (Habl and Auersperg 2017). Since male zebra finches are responsible for selecting and placing nest materials, choosing pieces that fit into their developing nests (Muth and Healy 2014), they may be more adept at learning about and evaluating object relationships due to evolutionary pressures associated with nest building. We employed two variations of the shape‐frame matching task, called the trap‐gap test, where birds pulled food‐containing trays attached to strings through gaps in barriers. In the barrier discrimination task, both trays were identical in size, but only one of two differently sized gaps allowed the tray to pass through. In the tray discrimination task, the gaps were the same size, but the trays varied, with only one small enough to fit. Half the birds were trained on the barrier discrimination task first, while the other half began with the tray discrimination task. To assess whether birds had learned a general rule—evaluating tray size relative to the gap—they were then transferred to the alternate task, hereafter referred to as the “transfer test.” Based on the nest‐building role of males, we predicted that male zebra finches would reach learning criteria more quickly and make fewer errors on the transfer test compared to females.

## 2 Methods

### 2.1 Subjects

37 adult zebra finches (20M; 17F) were bred and raised at the University of Alberta. Each bird hatched in our laboratory in King Cages (50 × 100 × 50 cm; King Cages International LLC) and was separated from its parents at nutritional independence (~35 days post‐hatch) into a cage with other juvenile birds. When the sex of each bird was distinguished via plumage (~45 days post‐hatch) it was moved into a same‐sex colony cage (165 × 66 × 184 cm). Fifteen birds, all in the Tray Discrimination Group (see details below) were then moved to a mixed‐sex aviary (457 × 305 × 244 cm); two birds in the Tray Discrimination Group were only housed in the aviary as adults. For birds housed in same‐sex colony cages, both male and female cages were housed in the same room and the birds had vocal and visual contact with the opposite sex. Birds in neither the colony cages nor the aviary had the opportunity to interact with string outside of trials; therefore, it is unlikely that rearing background influenced performance in string‐pulling tasks. Colony cages and the aviary were provided ad libitum access to water, mixed seed (Hagen Canada, Quebec, Canada), cuttlefish shell, oyster shell (Canadian Lab Diets Inc., Alberta, Canada), and gravel (Hartz, Ontario, Canada). Spray millet was provided once per week and spinach and Prime Vitamin Supplement (Hagen) were provided three times a week. The colony rooms and aviary were on a 14:10 light: dark cycle (full spectrum lights—Standard, 32W, T8 Daylight) with temperatures ~21°C and humidity ~40%. All care and experimental procedures were approved by the University of Alberta Animal Care and Use Committee (AUP 00002923).

### 2.2 Apparatus

**FIGURE 2:**
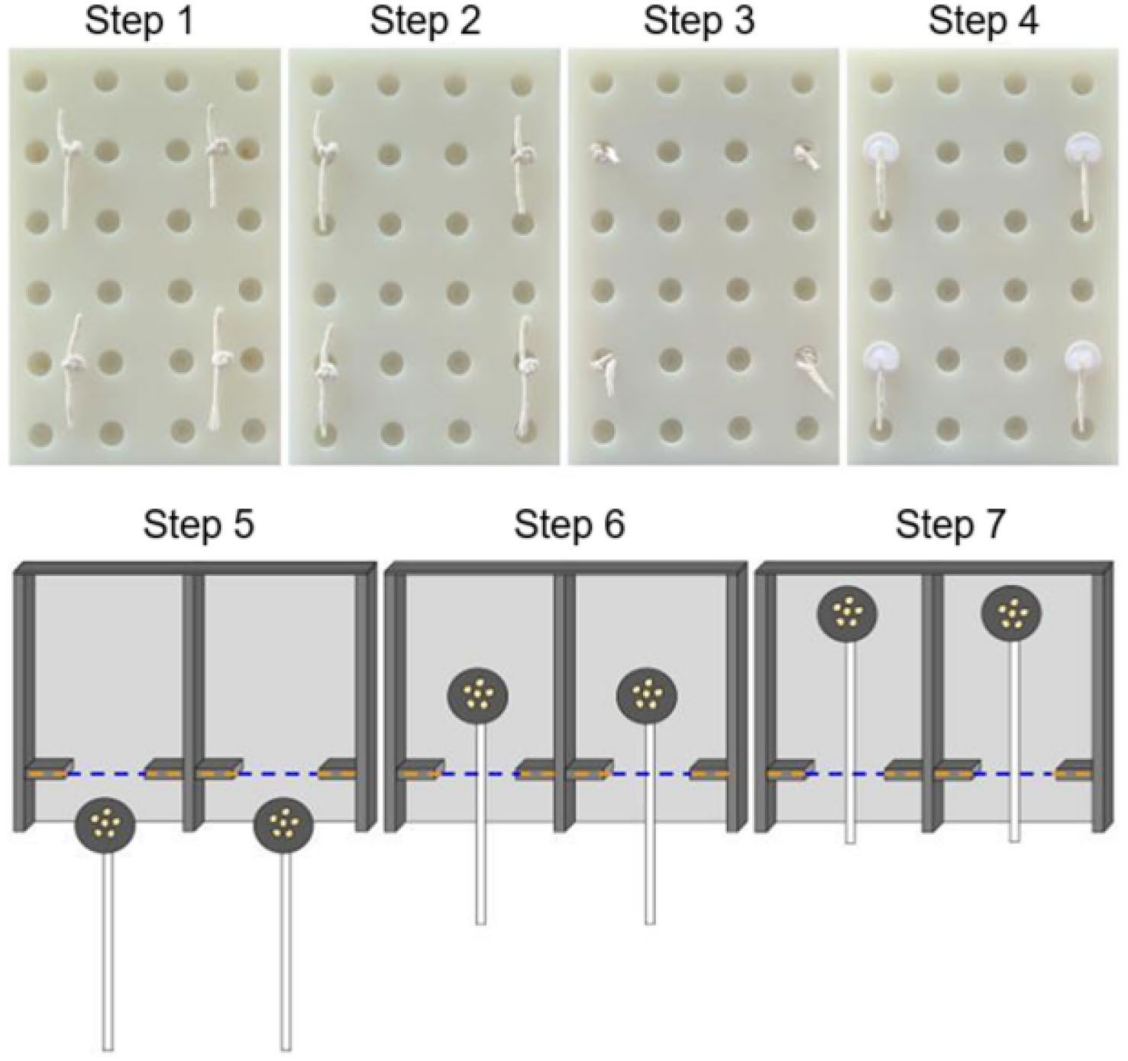
Apparatus and procedure for the seven different steps during shaping. Top row contains top‐down photos of the well‐board (21.4 × 14.5 cm), string and chips used for steps 1–4 of shaping. Bottom row are top‐down schematic views of the same‐barrier two‐choice boards (17.5 × 23.5 cm) and trays used for steps 5–7 of shaping. Seeds are shown by yellow circles, and are always in the same four wells in steps 1–4 and in both trays in steps 5–7. The blue‐dashed line highlights the gap (8.4 cm) on the board where the tray (3.5 cm) can be pulled through, with the barriers (1.5 cm) marked by yellow‐dashed lines.

During the experiment, each bird was housed in a same‐sex pair in King Cages with the same light: dark, temperature, humidity, food and supplements as in the colony and aviary housing. Behavior was recorded with a mini‐BNC camera (OSY CAMS) attached to the inside of the cage to the ceiling. The goal of shaping was so that birds learned how to use the apparatus before the critical physical cognition tasks (i.e., the Tray Discrimination and Barrier Discrimination, see Supporting Information: video). In steps 1–4 of shaping, we used a white plastic board (hereafter “*well‐board*”; 21.4 × 14.5 cm, Figure 2, top row) with 24 wells (1.3 cm diameter × 1.3 cm deep) arranged in a 6 × 4 grid, a 5 cm piece of white polished cotton string (James Leaver CO., Bristol, UK) with a knot tied in its center, opaque white plastic chips (1.9 cm diameter) with a piece of 2.5 cm long string attached via hot glue and a rubber stopper (5.0 mm deep) on the bottom that fit securely into the wells on the board. During steps 5–7 of shaping and for the cognitive tasks we used a series of plastic 17.5 × 23.5 cm two‐choice foraging boards (hereafter “*two‐choice boards*,” Figure 2, bottom row). The two‐choice boards had a white bottom with black walls and barriers that were 3.0 mm thick and 1.0 cm tall; these walls surrounded three of the four ends, with one of the long ends open and divided into two halves. A plexiglass cover was placed over the board to allow birds to see the rewards but prevent access to the items placed on the board except through the open ends. We used 3D‐printed black petri dishes of various sizes to hold millet seeds. These petri dishes (hereafter “*trays*”) had white strings attached to one end and were placed on the board underneath the cover. This setup required birds to pull the string at the end to remove the tray from under the plexiglass cover and access the food.

During steps 5–7 of shaping, each open half of the board had 1.5 cm length barriers and an 8.4 cm gap (Figure 2, hereafter “*shaping board*”) and a 3.5 cm tray with 12 cm string (Figure 2, bottom row).

**FIGURE 3:**
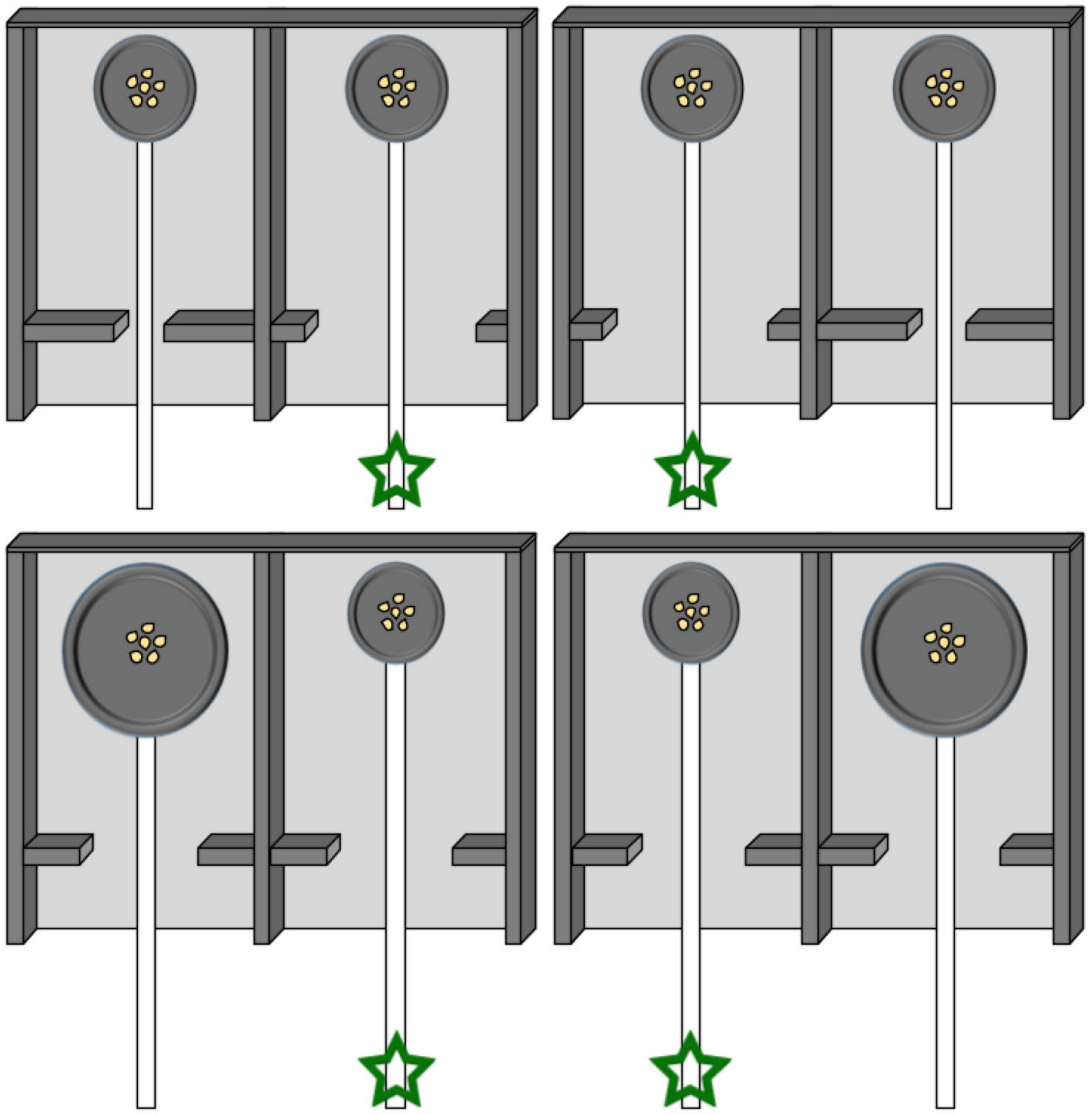
Schematics of the two‐choice foraging boards (17.5 × 23.5 cm) used in the barrier discrimination (top row) and the tray discrimination (bottom row). There were two boards used for each discrimination that had the S+ (accessible food) and S− (inaccessible food) on opposite sides. The green star indicates the correct choice for each board that allowed the bird to access the food. The trays in the barrier discrimination (top row, left board) are 4.0 cm diameter with 4.5 cm barriers and a 2.0 cm gap and 2.5 cm barriers and a 6.0 cm gap. In the tray discrimination (bottom row, left board) the tray on the left is 5.0 cm in diameter and the tray on the right is 3.0 cm in diameter and the barriers are 3.5 cm and the gaps are 4.0 cm. The boards on the right represent the same dimensions, but on opposite sides.

The two‐choice boards used during barrier discrimination had 4.0 cm diameter trays with a 14.0 cm piece of string glued to one end. On one side of the board the barriers were 2.5 cm long with a 6.0 cm wide gap, large enough for the 4.0 cm tray to fit through. The other side had 4.5 cm long barriers with an opening of 2.0 cm. Here, the bird could only access the food reward by pulling the string on the side of the board with the 6.0 cm gap (Figure 3, top). The two‐choice boards used during the tray discrimination had 3.5 cm long barriers and a 4.0 cm gap. One side had a 3.0 cm diameter tray with a 15.0 cm white string, and the other side had a 5.0 cm diameter tray with a 13.0 cm white string attached. Here, the bird could only access the food reward by pulling the string on the side of the board with the 3.0 cm diameter tray (Figure 3, bottom).

### 2.3 Cognitive Tasks

Each bird first underwent four steps of shaping with the well‐board following methods similar to (Boogert et al. 2008; Guillette et al. 2015; Lambert et al. 2021, 2022), and then went through three additional shaping steps with the two‐choice board (see Supporting Information: video and shaping section, below, for details). Each bird was then tested on a discrimination task followed by a transfer test following procedures similar to van Horik and Emery (2016). Half of the birds were trained in each of the tasks then transferred to the other task. On each training day, 2 h after lights switched on, the food cups were removed from each cage, and an opaque plastic divider was placed in the middle of each cage to separate paired individuals so they could be tested independently. The bottom of the cage was replaced (by sliding it out) with a clean cage bottom to prevent the birds from eating any spilled food. Ninety minutes after food removal, training trials began. Each trial lasted up to 3 min and was followed by a 12‐min inter‐trial interval; birds received 15 trials per day, each day of the week. Note that placing the foraging board into the cage only required sliding out and then replacing the cage bottom, rather than directly placing the board in the cage by hand.

#### 2.3.1 Shaping

We shape‐trained birds to pull strings to access food (millet seeds) using the well‐board (3 millet seeds in each well), and then continued to shape‐train the birds to pull strings on the two‐choice board to access food (6 millet seeds in each tray). Shaping consisted of a habituation phase, to habituate birds to the foraging boards and being separated into their own side of the cage for independent testing, followed by seven steps of shaping trials. During the habituation phase, two foraging boards (i.e., the well‐board and one of the two‐choice boards) were covered with millet seed and placed in the center of the cage bottom and the plastic divider was placed in the center of the cage to separate the birds into their half of the cage for 24 h prior to step 1 of shaping. See Supporting Information for video of birds completing each shaping and training step.

For the first four steps of shaping, each bird had to eat all millet seeds from three of the four baited wells to pass the trial, and the criterion to move on to the next step of shaping was passing three trials in a row, with trials cumulative across days. Each trial ended after 3 min, or once a bird ate all the food in all four baited wells, after which the board was removed from the cage. The same four wells were baited each trial. The goal was to train the birds to pull the string to access food.

In step 1 of shaping, three millet seeds were placed into four wells of the well‐board and a piece of 5.0 cm white string with a knot tied in the middle was placed next to each well but did not cover the wells. In step 2 each string was placed so that it now crossed the well, partially covering it. In step 3, the knot of the string was placed into the well so that the bird had to peck the string out of the way to access the food. In step 4, the string was attached to a chip with a plastic bumper and was placed in the well so that it required birds to physically pull the string to access food. If a bird did not access food for five consecutive trials during step 4, it was placed on a modified step where the chips with bumpers were tilted upwards, so that the food was visible but the birds had to move the chip out of the way to access the food. Once a bird passed three trials of this modified step it proceeded back to step 4.

Once a bird reached criterion in step 4 of shaping, it proceeded to shaping with the two‐choice board. For shaping steps 5–7 and all subsequent discriminations with the two‐choice boards, the board was rotated randomly each trial such that the open end of the board faced one of four directions relative to the front of the cage to ensure birds were not learning a rewarded spatial location but rather the affordances of the task. The board was never in the same direction for more than three consecutive trials. For each step of shaping with the two‐choice board, the bird had to eat from both trays for three consecutive trials to move to the next step. In step 5 of shaping, the trays (35 mm diameter) were placed at the edge of both sides of the board such that birds could pull the string or pull the tray itself to access the food. In step 6, the trays were placed in the middle of the board, and birds had to pull the string to access the food. In step 7 of shaping the tray was placed near the back of the board such that birds had to use multiple pulls of the string to access the food. Once birds passed step 7, they proceeded directly to the discrimination learning and then the transfer test.

#### 2.3.2 Discrimination and Transfer Test

Two boards each were used for both the barrier discrimination and the tray discrimination that had the food rewarding tray (S+) and non‐rewarding tray (S−) on opposite sides, and the board used on a given trial was semi‐randomly determined such that the S+ (the wider gap/smaller barriers that allowed the food tray to pass through) was never on the same side of the board/the same board used more than three consecutive trials. A bird's choice was recorded based on when it touched either string with its beak.

Half the birds first were trained to learning criterion on the barrier discrimination task (Barrier First Group) then transferred to the tray test for 15 trials. The other half of the birds first were trained to learning criterion on the tray discrimination task (Tray First Group) then transferred to the barrier test for 15 trials. Once a bird chose the S+ first for 10/12 trials in a row (Ashton et al. 2018; Shaw et al. 2015), indicating above‐chance performance, it passed the discrimination task (Figure 3). A bird was given a “null” trial if it did not make a choice in the 3‐min trial time, and these trials did not count towards birds' learning scores. After passing the discrimination task, birds were given all 15 trials of the transfer test the following day, after which they were returned to the colony room.

### 2.4 Statistical Analyses

We analyzed data using R, version 4.4.2 (R Core Team 2021). Significance was defined as *p* < 0.05 and data were visualized using the *ggplot2* package (Wickham et al. 2016). Generalized linear models (GLM) and generalized linear mixed models (GLMM) were fit using the *lme4* and *lmerTest* packages (Bates et al. 2015; Kuznetsova et al. 2017) with model diagnostics run in the *performance* package (Lüdecke et al. 2021). Diagnostics included checking models for over dispersion, influential outliers, zero inflation, collinearity, singularity, assessing model convergence, and comparing goodness of fit between models. A total of 37 birds began the experiment; though two males died due to unknown reasons before completing the discrimination tasks. One male failed to finish shaping and was excluded from further testing. Therefore, our final sample size was 34 birds (*n* females = 17, barrier task first = 9, tray task first = 8; n males = 17, barrier task first = 8, tray task first = 9).

We ran a Wilcoxon Rank Sum test to compare the number of trials taken to pass shaping steps 4–7 (initial learning to pull string/use the two‐choice board) between birds in the tray‐first and barrier‐first groups, thereby testing whether any existing differences (e.g., related to rearing environment) affected their baseline ability to complete discrimination tasks. Given our sample size and that data were not normally distributed, we ran four additional Wilcoxon Rank Sum tests to compare differences between: (1) ages of females and males, (2) ages of birds initially completing the tray/barrier task, (3) null trials of males and females, and (4) null trials of birds initially completing the tray/barrier task. To evaluate whether initial discrimination task (tray‐first/barrier‐first) or age (days) was a stronger predictor of learning during the discrimination tasks, we compared the AICc values of two GLMs, both of which used the number of trials to reach learning criterion as the response variable and one predictor of interest (initial task or age) as a fixed term.

To assess differences in learning among birds during the discrimination tasks, we ran a GLMM fit with a Poisson distribution that used the number of trials taken to reach criterion as our response variable. Birds that did not reach criterion (*n* males = 3, *n* females = 1) were given a score of 232 trials, which was the maximum number of trials permitted during the task. We included fixed terms for sex (female, male), initial discrimination task (tray, barrier) and an interaction term between the two factors to assess the performance of each sex across tasks. To account for variance in age and the number of null trials, we binned these variables into categorical factors (age bins = every 5 days, null trial bins = every 2 trials) and included these as random factors in the model, which avoided overfitting and aided model convergence. We re‐ran the same model using a dataset which excluded the four birds that did not reach criterion, checking whether omitting these individuals changed the model outcome.

To better assess the impact of birds not reaching criterion on the overall learning performance during tasks, we ran a time‐to‐event (survival) analysis. We fit and visualized a Cox proportional hazards regression model using the *survival* and *survminer* packages (Kassambara et al. 2024; Therneau 2024). The model defined time as the number of trials to reach criterion and event as whether the bird did (1) or did not (0) pass the discrimination task. We included fixed terms for initial task (barrier, tray) and sex (female, male). The relative risk calculated by the model therefore represents the risk of a bird failing the discrimination task, with a lower risk indicating fewer birds failed/more birds passed the task in a given number of trials.

To determine whether learning in discrimination tasks affected performance on the transfer test, we ran a GLM that used the number of errors a bird made during their transfer test as a response variable. We included fixed effects for sex (female, male), initial discrimination task (tray, barrier), and the number of trials taken to reach learning criterion during the discrimination task. Again, we re‐ran the same model and excluded data from the four birds which did not reach criterion during the discrimination task to assess whether omitting these individuals changed the model outcome. There were no null trials during the transfer test.

## 3 Results

There was no difference in the time taken to pass shaping procedures (*W* = 148.5, *p* = 0.904) between birds in the tray‐first group (median shaping trials = 17, min = 10, max = 91) and barrier‐first group (median shaping trials = 17, min = 10, max = 50), suggesting all birds began learning about the trap‐gap tasks from a comparable baseline.

Birds passed the discrimination tasks in a mean number of 111 ± SD 66 trials (median trials = 98, min = 23, max = 232). The overall proportion of null trials during discrimination tasks was low (2%, 79 of 3800 trials); however, females had significantly more null trials than males across both tasks (*W* = 205, *p* = 0.030; female null trials: mean = 3 ± SD 2, median = 3, min = 0, max = 8; male null trials: mean = 2 ± SD 4, median = 0, min = 0, max = 13). There was no significant difference in the number of null trials between birds that started with the tray or barrier discrimination task (*W* = 188.5, *p* = 0.116; tray‐first null trials: mean = 1 ± SD 2, median = 1, min = 0, max = 5; barrier‐first null trials: mean = 4 ± SD 4, median = 3, min = 0, max = 13).

There was no age difference between the female and male zebra finches (*W* = 133, *p* = 0.705; female age (days): mean = 660 ± SD 332, median = 864, min = 243, max = 1050; male age (days): mean = 687 ± SD 281, median = 528, min = 397, max = 1142). However, birds in the Tray First Group were younger than the birds in the Barrier First Group (*W* = 0, *p* < 0.001; tray‐first age (days): mean = 385 ± SD 82, media*n* = 413, min = 243, max = 528; barrier‐first age (days): mean = 963 ± SD 75, median = 979, min = 850, max = 1142). Comparing AICc values of two GLMs both fit with a single fixed effect revealed that initial task (AICc = 1117) was a stronger predictor of task performance than age (AICc = 1228).

**FIGURE 4:**
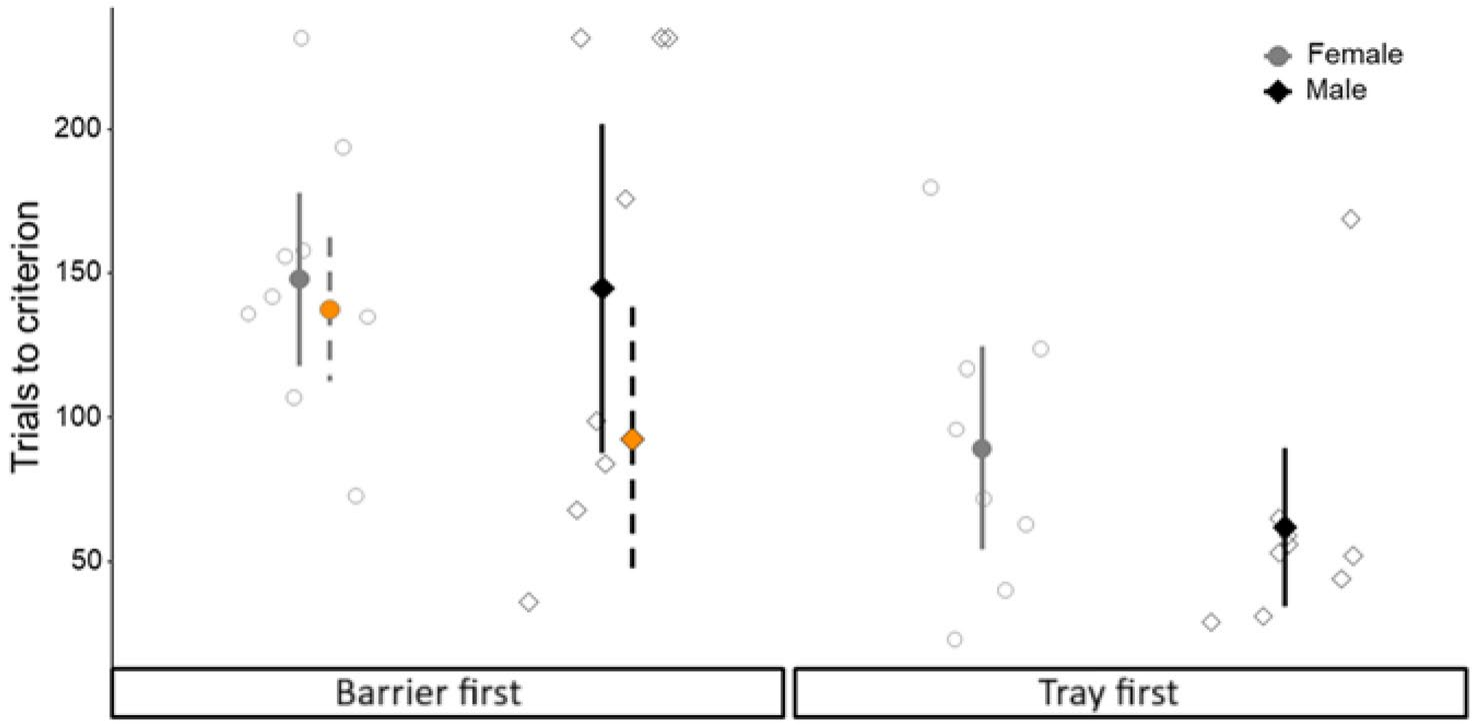
Trials to criterion (*y*‐axis) for birds trained in the barrier first group (left; *x*‐axis) and tray first group (right; *x*‐axis). Each open marker represents one individual and group means and 95% confidence intervals are represented by the filled markers (circles = female; diamonds = males). Orange points with dashed error bars in the barrier discrimination represent means and confidence intervals excluding the birds that failed this task (the four points at the top of the figure).

**FIGURE 5:**
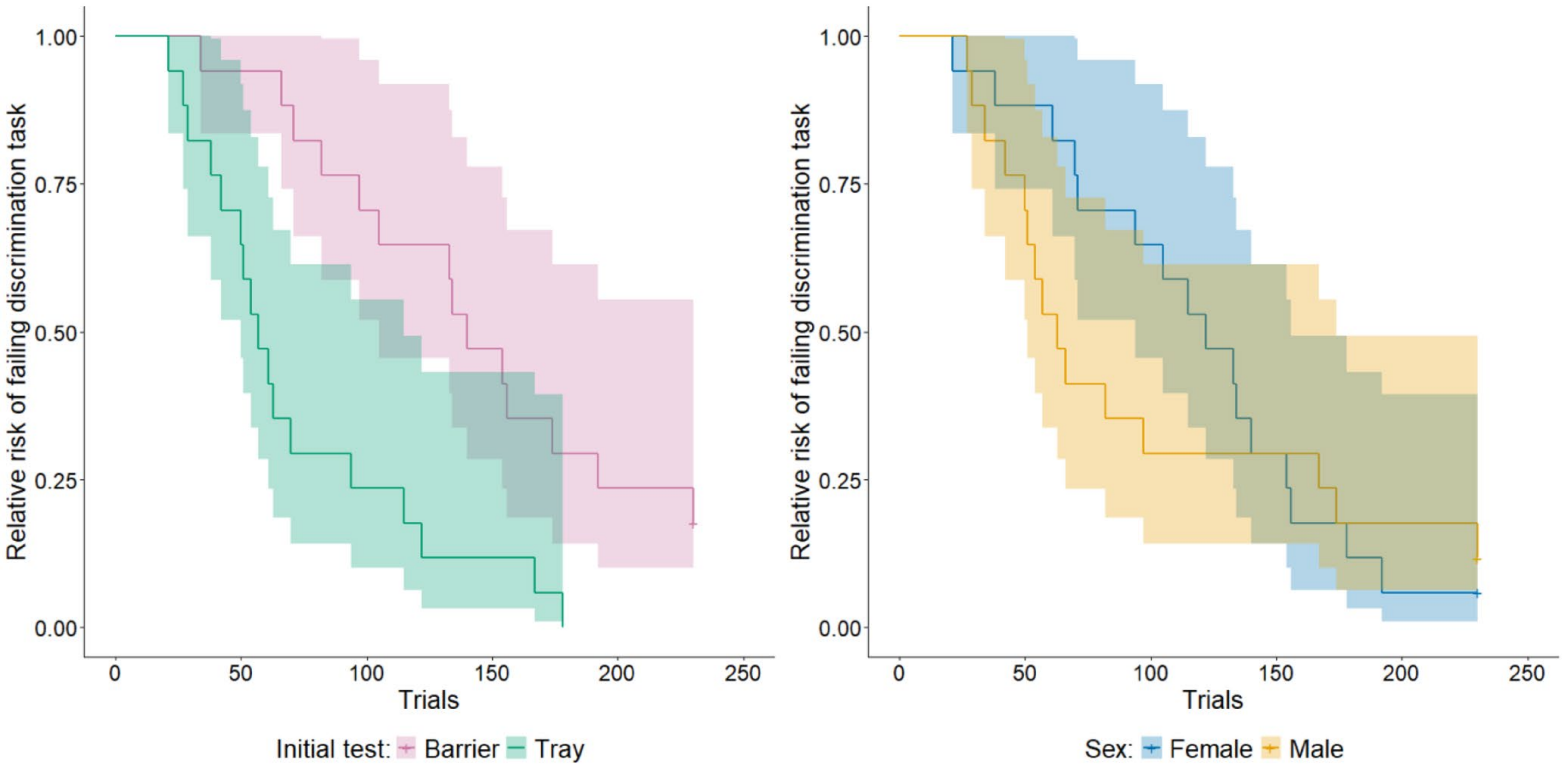
Relative risk of a bird failing the discrimination task over successive trials. The horizontal line drops down each time a bird reaches learning criterion. Leftmost plot compares relative risk between birds that started with the barrier task (purple) and tray task (green), and the rightmost plot compares relative risk between females (blue) and males (yellow). The shaded areas show 95% confidence intervals. On the left graph, 50% of the birds in the Tray First Group learned in just over 50 trials, but it took around 150 trials for 50% of the birds in the Barrier First group to learn.

There was no difference in the outcome of GLMMs that excluded or included birds which failed the discrimination task (*n* = 4), though the exclusionary GLMM had a better fit so we report results from this model. The number of trials taken to reach the learning criterion was not affected by sex (estimate = 0.004 ± SE 0.173, *z* = 0.002, *p* = 0.982) the initial task (estimate = −0.133 ± SE 0.270, *z* = −0.493, *p* = 0.622) or the interaction between sex and initial task (estimate = −0.327 ± SE 0.328, *z* = −0.997, *p* = 0.319, Figure 4). The log‐rank test performed by the Cox proportional hazards model was significant (*χ* ^2^ = 10.900, df = 2, *p* = 0.004), indicating there was a difference among birds in their relative risks of failing the discrimination task (Figure 5). Birds that were initially tested on the tray discrimination task were 3.6 times (95% confidence interval: 1.7–7.8) more likely to pass the learning criterion than birds initially tested on the barrier discrimination task, while adjusting for sex, with this difference being significantly different from zero (*z* = 3.257, *p* = 0.001). Whereas, males were 1.3 times (95% confidence intervals = 0.6–2.7) more likely to pass the learning criterion than females, while adjusting for their initial task, but this difference was not significantly different from zero (*z* = 0.683, *p* = 0.495).

**FIGURE 6:**
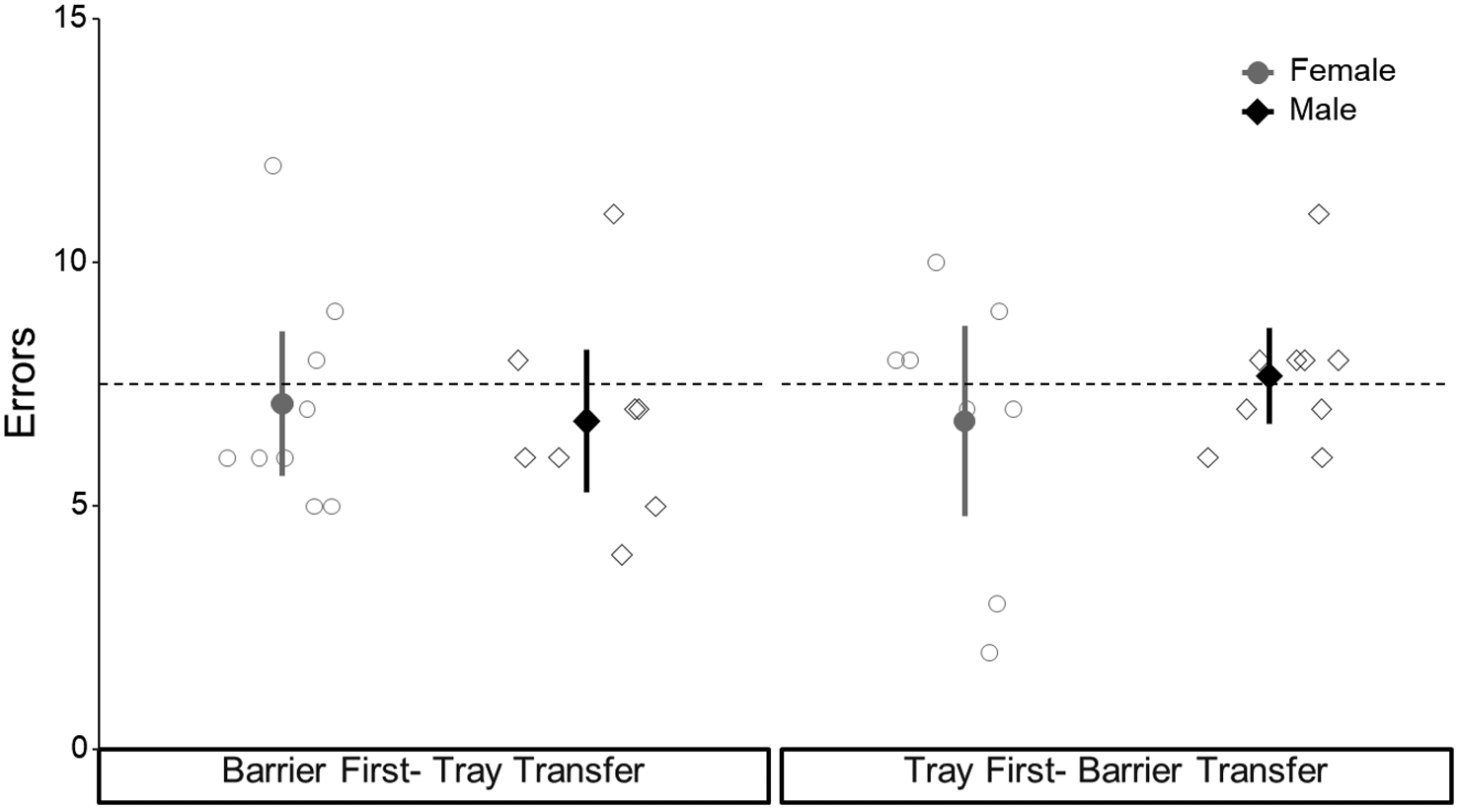
Number of errors (*y*‐axis) in the transfer test (*x*‐axis) for birds trained in the Barrier First (tray transfer) and Tray First Group (barrier transfer). Each open marker represents one individual and group means and 95% confidence intervals are represented by the filled markers (circles = female; diamonds = males).

The number of errors made during the transfer test was similar between the sexes (female errors: mean = 7 ± SD 2, median = 7, min = 2, max = 12; male errors: mean = 7 ± SD 2, median = 7, mi*n* = 4, max = 11, Figure 6) and among birds that began with different discrimination tasks (tray‐first errors: mean = 7 ± SD 2, median = 8, min = 2, max = 11; barrier‐first errors: mean = 7 ± SD 2, median = 6, min = 4, max = 12). There was no difference in the outcome of GLMs that included or excluded birds that failed to reach the learning criterion during the discrimination task (*n* = 4), so we report the exclusionary model as it had a better fit. There was no effect of sex on the number of errors made during the transfer test (estimate = 0.113 ± SE 0.147, *z* = 0.768, *p* = 0.442), and neither a bird's initial discrimination task (estimate = −0.036 ± SE 0.151, *z* = −0.238, *p* = 0.812) nor trials taken to pass the discrimination task (estimate = 0.001 ± SE 0.002, *z* = 0.301, *p* = 0.764) were associated with the errors made during the transfer test (see Figure 6).

## 4 Discussion

Most birds learned to solve the trap‐gap problem, a string‐pulling version of the shape‐frame matching task; however, the tray discrimination task was generally easier to acquire than the barrier discrimination task. Contrary to our predictions that nest‐building males outperform non‐nest‐building females on these physical cognition problems, we found no sex differences in the number of trials required to reach learning criteria. Performance at chance levels on the transfer tests suggests that birds did not solve initial discriminations by comparing the size of the tray to the size of the gap. Although females had more null trials than males, perhaps indicating a difference between sexes in their likelihood of engaging with the trap‐gap task, the overall proportion of null trials across the experiment was very low (2%) which limits the strength of any inference drawn from this result.

If the birds learned to solve either discrimination task by comparing the size of the trays to the size of the gaps, we would expect them to perform above chance on the transfer test. However, our data do not support this type of learning. Rather, in the barrier discrimination task, the birds learned to solve the task by selecting either: (1) the large gap, (2) the small barriers, (3) the larger gap, or (4) the smaller barriers. The first two options represent absolute strategies, while the latter two are relative strategies. In the tray discrimination task, the birds learned to solve the task by selecting either: (1) the small tray (an absolute strategy), or (2) the smaller tray (a relative strategy). While age was included as a random factor in our models, it is important to note that birds in the Tray Task were younger than birds in the Barrier Task. The zebra finches found the tray discrimination task easier to learn, suggesting that the tray was a more salient cue compared to the gap or the barriers. This may be because the seed reward was in the trays and therefore captured the attention of the birds more so than other features (i.e., gap or barriers) of the apparatus. Additionally, the trays may have been more salient because they are larger than the barriers. Lastly, the difference in tray size could have provided an additional cue for the birds to learn: weight. The parrots tested in van Horik and Emery (2016) similarly found the tray discrimination easier to solve compared to the barrier discrimination.

Sex differences in behavior did not translate into the predicted sex differences in cognition: nest‐building male zebra finches did not solve a physical cognition problem faster than non‐building females. Previous experiments investigating learning and memory related to the physical properties of materials have also failed to find the predicted sex differences in zebra finches, that is, that nest‐builders are better at these tasks compared to non‐builders (Lambert et al. 2021). Similar findings have been reported in the auditory domain. A 2016 meta‐analysis of 14 published studies found that female zebra finches learned auditory discriminations faster than males (Kriengwatana et al. 2016). These findings suggest that the sex exhibiting more vocal production (i.e., males sing more) does not necessarily perform better in related cognitive assays, as females show better performance in vocal perception tasks.

Beyond zebra finches, examples from a range of species show that sex differences in behavior do not always correspond to differences in cognition, even though cognitive abilities are expected to evolve under selection pressure unique to an animal's environment (Shettleworth 2010). Male bumblebees (*Bombus* spp.) forage solely for themselves, while females forage both for their own food as well as to provide for the hive, but these behavioral roles are not linked to sex differences in learning in wild or commercially reared bees (Muth et al. 2021; Wolf and Chittka 2016). Similarly, male cuttlefish ( *Sepia officinalis* ) engage in intensive competition for mates that may result in larger home ranges and sex differences in travel distances and information use. Yet no differences have been found in the spatial learning ability of cuttlefish (Jozet‐Alves et al. 2008).

The trap‐gap problem was originally designed to test whether two species of parrot, despite lacking tool‐use abilities, could generalize learned physical cognition rules about object relationships. It was hypothesized that parrots could accomplish these physical cognitive tasks due to their large relative brain size and complex social behavior, traits they share with tool‐using great apes and corvids (van Horik and Emery 2016). Although not the primary focus of our current study, comparing the cognitive abilities of these non‐building parrots with those of nest‐building zebra finches, who have a smaller relative brain size (Olkowicz et al. 2016), offers valuable insights from an evolutionary comparative cognition perspective. All birds successfully learned the tray discrimination task (parrots, *n* = 4; zebra finches, *n* = 17), which proved easier than the barrier discrimination task. Only half of the parrots and more than 75% of zebra finches learned the barrier task within the allotted number of trials (200 trials maximum for parrots; each of the zebra finches that learned the barrier task did so within this number of trials). These data suggest that the barrier discrimination task is more difficult for both species, despite their notably different ecologies and taxonomies. Notably, only one bird, a black‐headed caique ( *Pionites melanocephala* ) performed above chance on the barrier transfer test, suggesting that this individual learned a relative rule to solve the tray discrimination task.

Animals employ two frames of reference when moving themselves or objects: the egocentric (body‐centered) and the allocentric (external/environment‐centered). The egocentric frame of reference is ontogenetically primary and is ingrained in the vestibular system, and in birds, additionally supported in the lumbosacral organ (Stanchak et al. 2020) and guided by gravity, body position and movement (Fragaszy et al. 2011; Pick and Lockman 1981). In zebra finches, once the domed roof on the species‐typical nest is built, males rely on an egocentric frame of reference to carry nest material held in their beak through the nest entrance. That is, they maneuver their body plus material as a single unit. Laboratory experiments show that upon initial exposure to nest boxes with varying entrance sizes, male zebra finches select appropriately sized material, suggesting an awareness of the size relationship between the material and the nest entrance, through which they must carry that material. With experience, they learn how to manipulate all material such that it can fit into the nest entrance, regardless of size (e.g., bringing long, stiff material through a small opening; Muth and Healy 2014). In contrast, the trap‐gap test, engages an allocentric frame of reference, requiring the birds to pull a string attached to a tray (one external object), through a gap in a barrier (another external object); notably, both objects are external to the bird (Potì 2000). This mismatch in frames of reference—egocentric in nest building versus allocentric in the trap‐gap—may obscure potential sex differences in physical cognition in zebra finches.

Shifting the frame‐of‐reference from allocentric to egocentric leads to improved performance in trap‐table problems among tool‐using great apes (Girndt et al. 2008; Povinelli 2000; Seed et al. 2009). These and related problems such as the trap‐barrier (Martin‐Ordas et al. 2008) and trap‐tube (Seed et al. 2006) are designed to test physical cognition by requiring subjects to obtain food using a tool while avoiding a trap or obstruction. When tools were prepositioned on a platform and apes had to choose the correct one, they made more errors than when they were first given a tool and then allowed to choose how to solve the task (Girndt et al. 2008). Similarly, chimpanzees perform better on the trap‐tube problem when they use their finger, rather than a tool, to move food to an accessible location (Seed et al. 2009). These findings suggest that framing tasks egocentrically can facilitate better performance. Applied to zebra finches, these findings suggest that testing in an egocentrically framed physical cognition task may enhance performance in males but not in females.

We found no sex differences in learning in a physical cognition task, despite sex‐specific roles in zebra finch nest‐building behavior. While females do not typically select, carry, or deposit material in the nest, they do sit in the growing structure and frequently manipulate it by tucking in or, less often, rejecting materials deposited by the male (Zann 1996). These interactions, though limited, may nonetheless exert sufficient selective pressure for females to learn about the physical properties of nesting material. An intriguing possibility is that female zebra finches may influence nest construction decisions more than previously appreciated, and therefore also need to learn about the physical properties of nest‐building materials. Beyond simply accepting or rejecting materials, females might be communicating approval or disapproval of their mate's nest‐building choices. For instance, West and King discovered that non‐singing female brown‐headed cowbirds played an active role shaping male songs. Females perform a visual wing‐flick display after some male songs. These songs, in turn, elicit copulatory postures in females that are in breeding condition (West and King 1988). This analogy suggests the need for females to learn about the physical properties of nest materials to then communicate with the male during nest construction. Detailed analyses of female behavior during nest building are needed to test the hypothesis that females actively shape male nest‐building behavior through specific social cues.

Lastly, it is worth considering the diversity of courtship behavior across Estrildid species, particularly the ancestral “straw‐display” (Soma 2018; Zann 1996). This courtship display, performed by either male and/or female depending on the species, involves holding material in the beak and can be accompanied by singing (in males) and dancing (in both sexes). Recent work shows that male red‐cheeked cordon‐bleus ( *Uraeginthus bengalus* ) and star finches ( *Neochmia ruficauda* ) prefer longer pieces of material over shorter ones when given the choice in the laboratory. Similar preferences have been observed in the wild for blue‐capped ( *U. cyanocephalus* ) and red‐cheeked cordon‐bleus (Soma et al. 2025). If males preferentially select longer items for display, it follows that females may also be capable of discriminating material length. This mutual sensitivity to material properties suggests that both sexes may be under selective pressure to attend to, and potentially learn about, the physical characteristic of object length. Although this courtship display is absent in zebra finches (Zann 1996), our findings suggest that discriminating among materials with different physical properties could be a conserved trait for both sexes across the Estrildid lineage.

## Author Contributions

**Connor T. Lambert:** conceptualization (equal), data curation (equal), formal analysis (supporting), investigation (equal), methodology (equal), project administration (supporting), supervision (equal), visualization (supporting), writing – original draft (equal), writing – review and editing (equal). **Benjamin A. Whittaker:** formal analysis (lead), supervision (supporting), validation (supporting), visualization (equal), writing – review and editing (supporting). **Brandon Neil:** data curation (equal), formal analysis (supporting), investigation (equal), project administration (supporting), validation (supporting), visualization (supporting), writing – original draft (supporting), writing – review and editing (supporting). **Cailyn Poole:** data curation (supporting), investigation (supporting), project administration (supporting), writing – review and editing (supporting). **Julia L. Self:** conceptualization (supporting), data curation (supporting), formal analysis (supporting), investigation (supporting), methodology (supporting), project administration (supporting), supervision (supporting), writing – review and editing (supporting). **Andrés Camacho‐Alpízar:** investigation (supporting), project administration (supporting), writing – review and editing (supporting). **Sara C. Blunk:** data curation (supporting), investigation (supporting). **Lauren M. Guillette:** conceptualization (equal), data curation (supporting), formal analysis (supporting), funding acquisition (lead), methodology (equal), project administration (lead), resources (lead), supervision (lead), writing – original draft (lead), writing – review and editing (lead).

## Ethics Statement

All protocols involving animals were approved by the University of Alberta Animal Care and Use Committee (AUP 00002923).

## Conflicts of Interest

The authors declare no conflicts of interest.

## Supporting information


**Table S1:** Table showing outcomes for generalized linear model (GLM) and generalized linear mixed‐model (GLMM).
**Data S1:** The data used in the survival analyses.
**Data S2:** All data used in all analyses except the survival analyses.
**Data S3:** Statistical codes used in the manuscript.
**Video S1:** Seven steps of shaping, tray discrimination task, and barrier discrimination task.
**Video S2:** Male zebra finch bringing nest material through the entrance hole of the partially built nest.

## Data Availability

All data and R codes are provided as Supporting Information.
