# Supplementary material for: No Sex‐Differences in Learning Trap‐Gap Problems in Zebra Finches
Source: Ecol Evol. 2025 Nov 10;15(11):e72440. doi: 10.1002/ece3.72440 (PMC12602269; doi:10.1002/ece3.72440)
Supplement: Supplementary file 1 — Table S1: Table showing outcomes for generalized linear model (GLM) and generalized linear mixed‐model (GLMM). Data S1: The data used in the survival analyses. Data S2: All data used in all analyses except the survival analyses. Data S3: Statistical codes used in the manuscript. Video S1: Seven steps of shaping, tray discrimination task, and barrier discrimination task. Video S2: Male zebra finch bringing nest material through the entrance hole of the partially built nest. [file ECE3-15-e72440-s001.zip › DataS3_R-script.R2.pdf]

```
#Clear previous data
rm (list = ls())
#
#install required packages
library(dplyr)
library(lme4)
library(lmerTest)
library(performance)
library(ggplot2)
library(ggpubr)
#
#Upload data, Please see 'Key' tab of data file for column descriptions
Physical_Cognition_Data <- read.csv("Research/Bird/Physical Cognition/Physical_Cognition_Data.csv",
na = 'NA')
View(Physical_Cognition_Data)
#
##Remove birds that died or were excluded##
Physical_Cognition_Data <- Physical_Cognition_Data %>%
  filter(Status != "Exclude") %>%
  filter(Status != "Died")
View(Physical_Cognition_Data)
#
##Descriptive Statistics##
#By sex
summary_stats_sex <- Physical_Cognition_Data %>%
  group_by(Sex) %>%
  summarize( mean_trial = mean(Initial_Nulls),
             sd_trial = sd(Initial_Nulls),
             med_trials = median(Initial_Nulls),
```

```
            min_trials = min(Initial_Nulls),
            max_trials = max(Initial_Nulls),
            mean_age = mean(Age),
            sd_age = sd(Age),
            med_age = median(Age),
            min_age = min(Age),
            max_age = max(Age))
summary_stats_sex
#
#By initial task
summary_stats_task <- Physical_Cognition_Data %>%
  group_by(Initial_Task) %>%
  summarize( mean_trial = mean(Initial_Nulls),
            sd_trial = sd(Initial_Nulls),
            med_trials = median(Initial_Nulls),
            min_trials = min(Initial_Nulls),
            max_trials = max(Initial_Nulls),
            mean_age = mean(Age),
            sd_age = sd(Age),
            med_age = median(Age),
            min_age = min(Age),
            max_age = max(Age))
summary_stats_task
#
##Subset data for wilcox comparisons##
#For females#
female_data <- Physical_Cognition_Data %>%
  filter(Sex == "F")
View(female_data)
```

```
#For males#

male_data <- Physical_Cognition_Data %>%

  filter(Sex == "M")

View(male_data)

#For tray first#

tray_data <- Physical_Cognition_Data %>%

  filter(Initial_Task == "Tray")

View(tray_data)

#For barrier first#

barrier_data <- Physical_Cognition_Data %>%

  filter(Initial_Task == "Barrier")

View(barrier_data)

#

#Run Wilcox rank sum test to compare shaping length between tasks#

wilcox.test(tray_data$Shaping2, barrier_data$Shaping2, paired = FALSE)

#No difference

#

##Compared differences in age and null trials between groups##

#Run Wilcox rank sum test to compare ages between males and females#

wilcox.test(female_data$Age, male_data$Age, paired = FALSE)

#No difference

#

#Run Wilcox rank sum test to compare ages between tray and barrier first birds#

wilcox.test(tray_data$Age, barrier_data$Age, paired = FALSE)

#Difference, tray birds are younger

#

#Run Wilcox rank sum test to compare null trials between males and females

wilcox.test(female_data$Initial_Nulls, male_data$Initial_Nulls, paired = FALSE)

#Difference, females have more nulls
```

```
#
#Run Wilcox rank sum test to compare null trials between tray and barrier first birds#
wilcox.test(barrier_data$Initial_Nulls, tray_data$Initial_Nulls, paired = FALSE)
#No difference
#
#Compare AICc values to identify strongest predictor of task performance
#Bird age
glmA <- glm(Initial_Trials ~ Age, family = 'poisson', data = Physical_Cognition_Data)
model_performance(glmA) #1228.037
#Initial task
glmB <- glm(Initial_Trials ~ Initial_Task, family = 'poisson', data = Physical_Cognition_Data)
model_performance(glmB) #1117.081
#
##Age and nulls are significant co-variates. Create bins for random factors##
# Find min and max ages
min_age <- min(Physical_Cognition_Data$Age, na.rm = TRUE)
max_age <- max(Physical_Cognition_Data$Age, na.rm = TRUE)
# Create 5-day bins for age classes
Physical_Cognition_Data$Age_5day_Bin <- cut(Physical_Cognition_Data$Age,
                                  breaks = seq(from = floor(min_age),
                                            to = ceiling(max_age) + 5,
                                            by = 5),
                                  include.lowest = TRUE,
                                  right = FALSE)  # left-inclusive bins
# Check distribution
table(Physical_Cognition_Data$Age_5day_Bin)
#
# Find min and max null trials
min_nulls <- min(Physical_Cognition_Data$Initial_Nulls, na.rm = TRUE)
```

```
max_nulls <- max(Physical_Cognition_Data$Initial_Nulls, na.rm = TRUE)

# Create 2-trial bins for null trials

Physical_Cognition_Data$Nulls_2trial_Bin <- cut(Physical_Cognition_Data$Initial_Nulls,

                                    breaks = seq(from = floor(min_nulls),

                                            to = ceiling(max_nulls) + 2,

                                            by = 2),

                                    include.lowest = TRUE,

                                    right = FALSE)  # left-inclusive bins

# Check distribution

table(Physical_Cognition_Data$Nulls_2trials_Bin)

# Check bins in data frame

View(Physical_Cognition_Data)

#Run GLMM on all data to assess learning during discrimination task

glm1 <- glmer (Initial_Trials ~ Initial_Task * Sex + (1|Age_5day_Bin) + (1|Nulls_2trial_Bin), family = 'poisson', data = Physical_Cognition_Data)

check_overdispersion(glm1) #no over dispersion

check_outliers(glm1) #no outliers

check_zeroinflation(glm1) # no zero inflation

check_convergence(glm1) # model converged

check_singularity(glm1) # no singularity

check_collinearity(glm1) # low correlation

model_performance(glm1)

summary (glm1) #no significant effects

#

#Subset birds that passed

pass_data <- Physical_Cognition_Data %>%

  filter(Status == "Pass")

View(pass_data)

#
```

```
#Run GLMM on passing birds to assess learning during discrimination task

glm2 <- glmer (Initial_Trials ~ Initial_Task * Sex + (1|Age_5day_Bin) + (1|Nulls_2trial_Bin), family = 'poisson', data = pass_data)

check_overdispersion(glm2) #no over dispersion

check_outliers(glm2)

check_zeroinflation(glm2) # no zero inflation

check_convergence(glm2) # model converged

check_singularity(glm2) # no singularity

check_collinearity(glm2) # low correlation

model_performance(glm2) # better fit than glm1

summary (glm2) #no significant effects

#

#Transfer error summary stats

summary_stats_errors <- Physical_Cognition_Data %>%

  group_by(Sex, Initial_Task) %>%

  summarize( mean_errors = mean(Transfer_Errors),

            sd_errors = sd(Transfer_Errors),

            med_errors = median(Transfer_Errors),

            min_errors = min(Transfer_Errors),

            max_errors = max(Transfer_Errors))

summary_stats_errors

#

#Run GLMM on all birds to assess learning during transfer task

glm3 <- glmer (Transfer_Errors ~ Initial_Task + Sex + Initial_Trials, family = 'poisson', data = Physical_Cognition_Data)

check_ovglmer()check_overdispersion(glm3) #no over dispersion

check_zeroinflation(glm3) # no zero inflation

check_singularity(glm3) # singularity

check_collinearity(glm3) # low correlation
```

```
model_performance(glm3)
summary (glm3) #no significant effects
#
#Run GLM on passing birds to assess learning during transfer task
glm4 <- glm (Transfer_Errors ~ Initial_Task + Sex + Initial_Trials, family = 'poisson', data = pass_data)
check_overdispersion(glm4) #no over dispersion
check_zeroinflation(glm4) # no zero inflation
check_convergence(glm4) # model converged
check_singularity(glm4) # no singularity
check_collinearity(glm4) # low correlation
model_performance(glm4)
summary (glm4) #no significant effects
#

###Survival Analysis###
#remove previous data
rm (list = ls())
#load required packages
library(readxl)
library(survival)
library(survminer)
library(ggplot2)
library(ggpubr)
#Upload data#
Survival_Data <- read_csv("Research/Bird/Physical Cognition/Survival_Analysis.csv")
Survival_Data <- Survival_Analysis
attach(Survival_Data)
View(Survival_Data)
#Create function for making clean looking graphs#
```

```
cleanup=theme(panel.grid.major = element_blank(),panel.grid.minor =
element_blank(),panel.background = element_blank(),axis.line = element_line((color="black")))

#

#Fit Cox Proportion Hazards Model#

coxph1<-coxph(Surv (Z_Score,Event) ~ Initial_Test + Sex)

cox.zph(coxph1) #Check proportional variance among groups, assumption are not violated

summary(coxph1)

#Create model fits for graphs

coxph2<-survfit(Surv (Z_Score,Event) ~ Initial_Test, data = Survival_Analysis)

coxph3<-survfit(Surv (Z_Score,Event) ~ Sex, data = Survival_Analysis)

#Make Plots

Fig1 <- ggsurvplot(coxph2, conf.int = TRUE,

           xlab = c("Trials"),

           ylab = c("Relative risk of failing discrimination task"),

           font.x = c(20),

           font.tickslab = c(18),

           font.y = c(20),

           legend = "bottom",

           palette = c("#CC79A7","#009E73"),

           font.legend = c(20),

           legend.title = "Initial test:",

           legend.labs = c("Barrier", "Tray"))

Fig1

Fig2 <- ggsurvplot(coxph3, conf.int = TRUE,

           xlab = c("Trials"),

           ylab = c("Relative risk of failing discrimination task"),

           font.x = c(20),

           font.tickslab = c(18),

           font.y = c(20),
```

```
        legend = "bottom",
        palette = c("#0072B2","#E69F00"),
        font.legend = c(20),
        legend.title = "Sex:",
        legend.labs = c("Female", "Male"))
Fig2
#Combine plots into one figure
splots <- list()
splots[[1]] <- Fig1
splots[[2]] <- Fig2
Survival_Figure<-arrange_ggsurvplots(splots, print = TRUE,
                ncol = 2, nrow = 1)
Survival_Figure
```
