# Supplementary material for: No Sex‐Differences in Learning Trap‐Gap Problems in Zebra Finches
Source: Ecol Evol. 2025 Nov 10;15(11):e72440. doi: 10.1002/ece3.72440 (PMC12602269; doi:10.1002/ece3.72440)
Supplement: Supplementary file 1 — Table S1: Table showing outcomes for generalized linear model (GLM) and generalized linear mixed‐model (GLMM). Data S1: The data used in the survival analyses. Data S2: All data used in all analyses except the survival analyses. Data S3: Statistical codes used in the manuscript. Video S1: Seven steps of shaping, tray discrimination task, and barrier discrimination task. Video S2: Male zebra finch bringing nest material through the entrance hole of the partially built nest. [file ECE3-15-e72440-s001.zip › Supplementary Table S1.docx]

|  |  |  |  |  |  |  |
| --- | --- | --- | --- | --- | --- | --- |
| **Model** | **Response** | **Effect** | **Estimate** | **±S.E.** | **z** | **P** |
| GLMM | Number of initial trials | Initial task | -0.133 | 0.270 | -0.493 | 0.622 |
|  |  | Sex | 0.004 | 0.173 | 0.002 | 0.982 |
|  |  | Initial task : Sex | -0.327 | 0.328 | -0.997 | 0.319 |
| GLM | Number of errors | Initial task | -0.036 | 0.151 | -0.238 | 0.812 |
|  |  | Sex | 0.113 | 0.147 | 0.768 | 0.442 |
|  |  | Number of initial trials | 0.001 | 0.002 | 0.301 | 0.764 |

Table S1. Table showing outcomes for Generalized Linear Model (GLM) and Generalized Linear Mixed-Model (GLMM), including the response term, fixed and interaction effects, estimates and standard error, z value and p value.
